# Estimating Indicators for Assessing Knee Motion Impairment During Gait Using In-Shoe Motion Sensors: A Feasibility Study †

**DOI:** 10.3390/s24237615

**Published:** 2024-11-28

**Authors:** Kazuki Ihara, Chenhui Huang, Fumiyuki Nihey, Hiroshi Kajitani, Kentaro Nakahara

**Affiliations:** Biometrics Research Labs, NEC Corporation, Hinode 1131, Abiko 270-1198, Chiba, Japan

**Keywords:** gait analysis, in-shoe motion sensor, foot motion, knee motion, stiff knee

## Abstract

Knee joint function deterioration significantly impacts quality of life. This study developed estimation models for ten knee indicators using data from in-shoe motion sensors to assess knee movement during everyday activities. Sixty-six healthy young participants were involved, and multivariate linear regression was employed to construct the models. The results showed that eight out of ten models achieved a “fair” to “good” agreement based on intra-class correlation coefficients (ICCs), with three knee joint angle indicators reaching the “fair” agreement. One temporal indicator model displayed a “good” agreement, while another had a “fair” agreement. For the angular jerk cost indicators, three out of four attained a “fair” or “good” agreement. The model accuracy was generally acceptable, with the mean absolute error ranging from 0.54 to 0.75 times the standard deviation of the true values and errors less than 1% from the true mean values. The significant predictors included the sole-to-ground angles, particularly the foot posture angles in the sagittal and frontal planes. These findings support the feasibility of estimating knee function solely from foot motion data, offering potential for daily life monitoring and rehabilitation applications. However, discrepancies in the two models were influenced by the variance in the baseline knee flexion and sensor placement. Future work will test these models on older and osteoarthritis-affected individuals to evaluate their broader applicability, with prospects for user-tailored rehabilitation applications. This study is a step towards simplified, accessible knee health monitoring through wearable technology.

## 1. Introduction

### 1.1. Background

The proper functioning of the knee joint is critical for individuals’ daily movement. Any physical abnormalities or injuries that impair knee motion can significantly impact one’s quality of life [1,2]. Elderly individuals are particularly susceptible to musculoskeletal issues such as knee osteoarthritis (OA), necessitating timely treatment and daily monitoring to prevent exacerbation [3]. Early-phase rehabilitation is crucial, as it assists physicians and clinicians in accurately identifying patients’ therapeutic needs, ultimately leading to better outcomes.

In healthy individuals, the knee flexion angle motion pattern (in the sagittal plane) reveals specific characteristics that change as the knee joint deteriorates [4]. Typically, a healthy flexion angle pattern showcases two peaks at 10% and 70% of the gait cycle (GC). However, with knee deterioration, this pattern alters, resulting in a shallower curve between these peaks and reduced peak heights at both 10% and 70% of the GC. Additionally, the position of the second peak within the GC shifts to a later phase. Monitoring these alterations can be helpful for the early detection of knee deterioration.

In the past decade, there has been a proliferation of wearable sensor systems designed to monitor these knee indicators for the early detection of knee motion impairment on a daily basis. For instance, Tao et al. [5] reported using accelerometers and gyroscopes embedded in wearable devices for knee movement and gait monitoring, with wireless transmitters attached to the upper and lower legs and feet. Togenetti et al. [6] utilized a flexible goniometer and accelerometer in a wearable setup to measure knee joint angles in everyday scenarios. Buttner et al. [7] integrated a potentiometer into a knee brace for continuous knee angle monitoring. Bolam et al. [8] employed multiple IMU sensors on the leg to track knee behavior following knee arthroplasty. Generally, these sensor systems are attached to the upper and lower legs to measure knee movement [9]. However, from the perspective of patient compliance, attaching bulky sensor systems to the leg can be inconvenient due to interference with clothing or daily activities. Reducing the weight and size of these sensor systems is technologically challenging and costly, necessitating the development of accurate, inexpensive, and user-friendly methods for assessing daily knee motion.

Recent advancements in Internet of Things (IoT) technology have enabled the implementation of wearable motion sensors for various healthcare applications, including gait analyses [10,11]. Smart wearable in-shoe motion sensors (IMSs) can be strategically placed in shoes or insoles to provide accurate foot motion analyses for everyday healthcare. Although IMSs do not directly measure knee movements, previous studies have demonstrated their effectiveness in detecting knee osteoarthritis (OA) gait features through foot motion analyses, even extending across the ankle joint [12]. This efficacy is likely due to the interconnected nature of lower limb locomotive actions and foot movement, also known as the “kinetic chain” [13]. When motion in the ankle or foot is restricted, it can impact the movement of adjacent joints, including the knee. This interconnectedness means that each segment of the kinetic chain can influence the performance and strain on the others. Additionally, muscle interplay involves the activation of specific muscle groups to stabilize or mobilize different parts of the body. If certain muscles in the foot or ankle are weakened or constrained, it can lead to an increased load or altered movement patterns in the knee, highlighting the importance of integrated movement within the kinetic chain [14]. Yang et al. [15] suggested that joint angle waveforms during gait can be estimated with the foot pressure and IMU sensors in a shoe via a machine learning approach. Therefore, we propose that it is feasible to estimate knee indicators using IMS-measured foot movement, which could be beneficial for individuals with knee issues during everyday walking. However, to the best of our knowledge, a study estimating knee motion indicators using only a single IMU on the foot has not been conducted. Therefore, this research challenges the feasibility of estimating knee motion during gait using an IMS.

Considering the limitation of computational resources on edge devices, rather than focusing on waveforms throughout the entire gait cycle, we focused on estimating intuitive and easily interpretable indicators directly related to knee joint abnormalities during walking. By reviewing previous biomechanical studies [4,16,17,18,19,20,21], in this feasibility study, we summarized ten quantitative indicators derived from the knee joint angle in the sagittal plane that are effective for evaluating knee motion impairment [17]. These ten indicators encompass four types of knee joint angle indicators, two types of temporal indicators [4,17,18,19,20,21], and four types of angular jerk cost (AJC) indicators [16]. The first six indicators reflect the static characteristics of knee motion at specific gait phases in one cycle, while the latter four reflect the dynamic characteristics of knee motion during gait. Detailed descriptions of these indicators are provided in Section 1.2. Our aim was to develop efficient models for estimating the ten indicators using only inertial foot motion data from an IMS.

Notably, this manuscript builds upon our previous research presented at IEEE I2MTC 2023 [22]. To ensure compatibility with edge devices, such as the IMSs used in healthcare applications, these models must be lightweight and interpretable. Our prior study involved constructing and evaluating estimation models for four knee angle indicators and two temporal indicators using our proposed statistical parametric mapping leave-one-subject-out least absolute shrinkage and selection operator (SPM-LOSO-LASSO) framework [23] and multivariate linear regression. This framework has demonstrated effectiveness in estimating various physical indicators, such as the foot function, muscle strength, and balance ability, using IMS-measured foot movement [22,23,24,25]. In this paper, we extend our framework to construct estimation models for the AJC indicators and broaden our scope by analyzing the correlation between foot motion and the total ten knee motion indicators.

### 1.2. Knee Indicators

**Figure 1:**
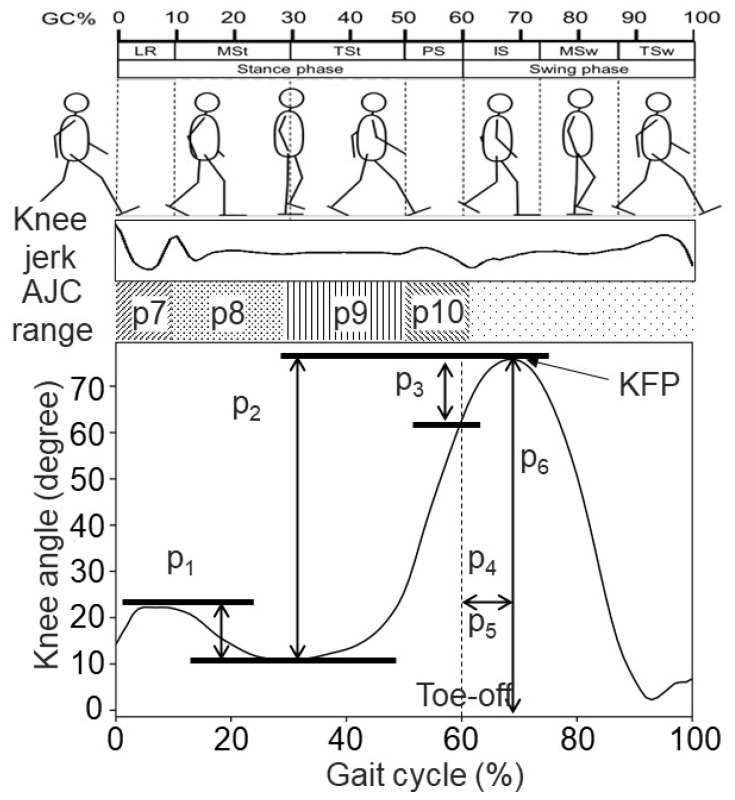
Knee motion features in one stride of knee flexion angle waveform (sagittal plane). p1 to p3 and p6 depict knee joint indicators; p4 and p5 depict temporal indicators; p7 to p10 depict AJC indicators. Blocks with different patterns in the upper side of the figure represent the region of interest in the gait cycle for calculating four types of AJC indicators, denoted as the AJC range. AJC: angular jerk cost; LR: loading response; MSt: mid-stance; TSt: terminal stance; PS: pre-swing; IS: intimal swing; MSw: mid-swing; TSw: terminal swing; KFP: knee flexion peak.

Figure 1 illustrates the selected indicators, designated as p1 to p10. Indicator p1 denotes the difference between the knee flexion angle values at 1% of the GC and 30% of the GC during the stance phase [6]. Indicator p2 represents the difference between the valley and the maximum knee flexion peak (KFP) [17,18,21]. Indicator p3 is the difference between the angle at toe-off and the maximum flexion angle [17,19]. Indicator p4 is the time duration from toe-off to the KFP, measured in seconds. Indicator p5 indicates the same time duration as p4, but expressed as a percentage of the swing phase [20], calculated using Equation (1):

(1) $$p5=\frac{t_{KFP}-t_{St}}{t_{Sw}}=\frac{p4}{t_{Sw}}$$

where *t_KFP_* and *t_St_* represent the time duration from the start of one stride to the KFP and toe-off, respectively. *t_St_* also signifies the stance phase’s time duration, while *t_Sw_* means the time duration of the swing phase. Lastly, indicator p6 represents the absolute angle at maximum flexion [17,20,21]. Typically, OA patients exhibit smaller values for indicators p1, p2, p3, and p6, and larger values for indicators p4 and p5.

Measurements of the jerk are proposed to objectively represent the smoothness of joint movement by calculating the time-dependent changes in acceleration [26]. The smoothness of movement is represented by the sum of the squares of the jerk (the jerk cost); a lower jerk cost value indicates smoother, more uniform speed movement [26]. The angular jerk cost (AJC) is computed by integrating the knee jerk waveform, which is expressed by Equation (2):

(2) $$AJC=\log\int_{t_{1}}^{t_{2}}{\left(\frac{{∆}^{3}\theta }{∆t^{3}}\right)}^{2}dt$$

where *t* represents time, *t*_1_ and *t*_2_ denote the start and end of the region of interest (ROI), and *θ* is the knee flexion angle during the gait.

In this study, the AJC is defined as the jerk cost of the knee joint angular movement in the sagittal plane. Previous studies have shown that subjects with more severe knee OA exhibit a lower AJC during all four periods of the stance phase—the loading response, the mid-stance, the terminal stance, and the pre-swing—compared to less affected patients [16,27]. Consequently, we calculated four indicators (p7 to p10) representing the knee AJC characteristics during the corresponding stance periods, as illustrated in Figure 1.

### 1.3. Main Contributions of This Study

The key contributions of this research include the following:

(1) An analysis of the correlation between ten indicators for assessing knee motion impairments and foot motion during gait.
(2) The development of lightweight linear estimation models for knee motion indicators predicated on foot motion, rendering them compatible for IMS implementation.

## 2. Materials and Methods

### 2.1. Participants

We collected data for the model construction from 66 participants (35 male and 31 female) with an age of 44.5 ± 15.9 years, a height of 164.5 ± 9.3 cm, and a weight of 61.4 ± 14.1 kg at the Biometrics Labs of the NEC Corporation. All the participants could walk independently without any assistive devices. They had normal or corrected-to-normal vision, no history of severe neuromuscular or orthopedic diseases, and no communication obstacles. We explained the experimental procedure to all the participants and obtained informed consent before the experiment. The study was approved by the NEC Ethical Review Committee for Life Sciences.

### 2.2. Experiments for Model Construction

#### 2.2.1. Data Collection

To collect knee behavior and foot motion data, the participants were asked to perform two round-trip walks of five meters in a straight line at a comfortable, self-determined pace. They walked on a surface covered with polyvinyl chloride carpet tiles. Each one-way segment constituted a single trial, resulting in a total of four walking trials. For analytical purposes, the initial and final steps of each trial were excluded.

**Figure 2:**
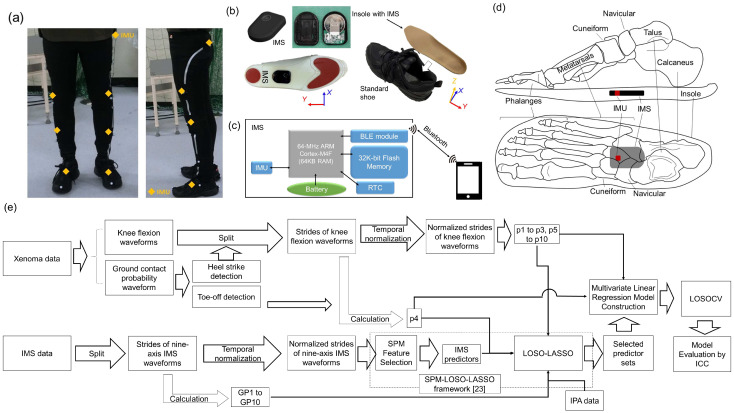
Experimental apparatus. (**a**) e-Skin MEVA pants worn by the subjects; yellow diamonds mean the location of IMUs on the pants. (**b**) IMS-settled in-sole and in-sole-installed sports shoes. *A_x_* (medial: +, lateral: −); *A_y_* (posterior: +, anterior: −); *A_z_* (superior: +, inferior: −); *G_x_*, *E_x_* (plantarflexion: +, dorsiflexion: −); *G_y_*, *E_y_* (eversion: +, inversion: −); and *G_z_*, *E_z_* (internal rotation: +, external rotation: −). (**c**) The circuits of the IMS, including a 6-axis IMU (BMI 160, Bosch Sensortec, Reutlingen, Germany), an ARM Cortex-M4F micro-control unit (MCU) (nRF52832, CPU: 64 MHz, RAM: 64 KB, ROM: 512 KB, Nordic Semiconductor, Oslo, Norway), an EEPROM (S-24C32C, 32K-bit, ABLIC, Tokyo, Japan), a real-time clock (RTC) (RX8130CE, EPSON, Suwa, Japan), and a 3-volt lithium-coin battery (CR2430, 300 mAh). The MCU included a Bluetooth low-energy (BLE) module. (**d**) Brief schematic of the location of the worn IMS and the IMU inside. (**e**) Brief flowchart of model construction and evaluation flow.

The knee angle in the sagittal plane was measured using sensor-embedded pants (e-skin MEVA, Xenoma Inc., Tokyo, Japan; see Figure 2a) worn by the participants during the walks. The pants were equipped with seven IMUs, each containing a three-axis accelerometer and a three-axis gyroscope, allowing for the estimation of three-dimensional joint movements and global positioning based on established algorithms [28]. The precision of the algorithm used for three-dimensional bone modeling in the e-skin MEVA has been validated in prior research and further corroborated by the results of our preliminary experiments, which benchmarked it against traditional motion capture systems [28]. The measurement datasets included unprocessed signals from IMU sensors (acceleration and angular velocity), the global position of each sensor and anatomical landmark, and the joint angles in the pelvis, hip, knee, and ankle, totaling 347 parameters. These datasets were automatically transmitted to a terminal PC via Bluetooth. The sensor data were transmitted to a personal computer via Bluetooth. The heel trajectory data and knee flexion angles in the sagittal plane were automatically output using software for e-skin MEVA (e-skin MEVA application, ver. 4.0.3, Xenoma Inc., Tokyo, Japan; denoted as Xenoma software hereafter), which subsequently served as training data for model construction.

For foot motion measurements, the participants wore sensor-embedded sports shoes. The same IMS described in Ref. [21] was used in this study (Figure 2b). This device, weighing 10 g, including the coin battery, and measuring 29 mm × 40 mm × 7 mm, was attached to the insole under the foot arch to ensure comfort during gait (see Figure 2d). For participants with a foot size of 24 cm, for instance, the IMU inside the IMS was approximately located under the intermediate cuneiform. The shoes fit tightly, modeling the mid-foot and hindfoot as a rigid body, thus allowing us to equate the IMS signals with foot motion signals. The IMS provided nine types of foot motion signals: three-dimensional acceleration (*A_x_*, *A_y_*, and *A_z_*), three-dimensional angular velocity (*G_x_*, *G_y_*, and *G_z_*), and the sole-to-ground angles (SGAs) of the roll, pitch, and yaw (*E_x_*, *E_y_*, and *E_z_*), calculated using a Madgwick filter inside the IMS [29]. During this feasibility study, detailed foot motion waveforms were recorded in real time by transferring them to a smartphone via the Bluetooth universal asynchronous receiver/transmitter (UART) mode (Figure 2c). Partial data loss occurred in the foot motion waveforms due to packet loss induced by limitations in the Bluetooth communication capacity and conditions. We derived the acceleration elements (*A_x_*, *A_y_*, and *A_z_*) in the global coordinates for each independent trial. The sampling rate was set at 100 Hz.

#### 2.2.2. Signal Processing

A simplified diagram of the model construction and evaluation process is presented in Figure 2e. Initially, heel strike events, expressed as ground contact probabilities (one of the parameters in the Xenoma datasets), were automatically synchronized with the other parameters using the Xenoma software. These probabilities were identified based on established algorithms [28]. Using Python 3.8, we partitioned the original knee flexion angle data in the sagittal plane into individual strides, defined as the interval from one heel strike to the next. In this study, the heel strike event of the Xenoma data was defined as the time point when ground contact probabilities exceeded a threshold of 0.95, while the toe-off event was defined as the time point when these probabilities fell below a threshold of 0.05. Indicator p4 was computed from the raw stride data based on the definition described in Section 1.2. The partitioned stride data were then normalized on a scale of 1 to 100% of the GC. Following normalization, the knee indicators p1 to p3 and p5 to p10 were computed for each stride from the normalized stride data.

After processing the knee flexion angle data, all the remaining data, the simulations, and the model construction were carried out in MATLAB (MathWorks, Natick, MA, USA). The original IMS signals underwent low-pass filtering with a 20 Hz cut-off frequency. The foot motions recorded by the IMS were divided into individual strides by identifying heel strike events through a previously constructed gait event detection algorithm for IMS [30]. These partitioned stride data points were temporally standardized between 1 and 100% of the GC.

Next, ten predictors for gait indicators in each stride were derived using IMS signals. These predictors included the stride length (GP_1_), gait speed (GP_2_), maximum SGA in the dorsiflexion direction (GP_3_), SGA in the plantarflexion direction (GP_4_), maximum circumduction (GP_5_), maximum foot height (GP_6_), toe in/out angle in the transverse plane (GP_7_), proportion of stance phase (GP_8_) and swing phase (GP_9_), and stride time (GP_10_). The stride length, maximum foot height, and maximum circumduction were normalized by the participant’s height during the calculations. The measured gait parameters are presented in Table 1.

#### 2.2.3. Model Construction and Evaluation

To construct models for p1 to p10, the SPM-LOSO-LASSO algorithm incorporated four types of variables: (1) individual physical attributes (IPAs), including sex (male: 0; female: 1), age, height, weight, and BMI; (2) ten temporospatial GPs [22], as described previously; (3) temporally normalized stride waveforms from the IMS; and (4) target variables (Figure 2e). To reduce noise, the average of all the GPs and IMS waveforms within each trial was calculated, resulting in one representative stride. A total of 403 strides were obtained in our dataset.

Using the SPM-LOSO-LASSO framework [23], significant gait phases related to the target variables were identified and classified as gait phase clusters (GPCs). Novel predictors, referred to as IMS predictors, were discovered, and sets of predictors were automatically optimized. The models were constructed using multivariate linear regression methodology, and leave-one-participant-out cross-validation (LOSOCV) was applied for model evaluation. The evaluation indices included a type (2, 1) intraclass correlation coefficient denoted as ICC (2, 1), the mean absolute error (MAE), the coefficient of determination (*R*^2^), Pearson’s correlation coefficient (r), and the mean value and standard deviation (SD). The guidelines for interpreting the ICC inter-rater agreement were as follows: excellent (>0.750), good (0.600–0.749), fair (0.400–0.599), and poor (<0.400) [31]. The interpretation guidelines for r were none (<0.100), small (0.100 to 0.299), medium (0.300 to 0.499), and large (>0.499), while those for *R*^2^ were none (<0.020), small (0.020 to 0.129), medium (0.130 to 0.259), and large (>0.259) [32].

## 3. Results

### 3.1. Statistical Analysis of Correlation Between Demographic Data and p1 to p10

Table 2 presents the results of the statistical analysis of the correlation between demographic data and indicators p1 to p10. A significant sex difference was found for all the knee motion indicators except for p3 and p6. The male participants tended to have stronger values in p1 and p7 to p10, but exhibited a weaker p2 and a shorter p4 and p5 compared to the female participants. Age was significantly correlated with all the knee motion indicators, particularly impacting p2, p4, p5, and p6 negatively, and p7 to p10 positively, with medium to large effect sizes. The BMI was significantly correlated with p1, p6, and p8 in a negative direction, and with p3, p5, and p10 in a positive direction; however, the effect sizes for these correlations were small.

### 3.2. Selected Predictors of Models p1 to p10

**Figure 3:**
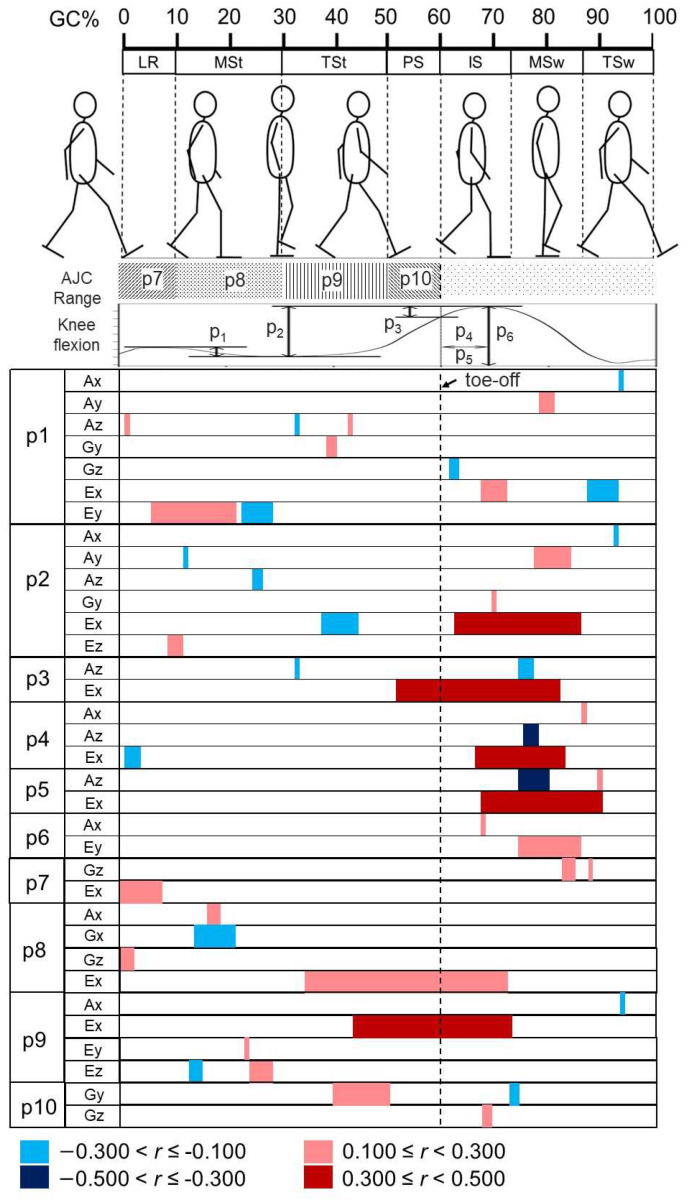
Schematic diagram of selected feature’s range of GPCs in each model; red blocks: positive correlation with target variables; blue blocks: negative correlation with target variables. LR: loading response; MSt: mid-stance; TSt: terminal stance; PS: pre-swing; IS: intimal swing; MSw: mid-swing; TSw: terminal swing. Biomechanical direction: *A_x_* (medial: +, lateral: −); *A_y_* (posterior: +, anterior: −); *A_z_* (superior: +, inferior: −); *G_x_*, *E_x_* (plantarflexion: +, dorsiflexion: −); *G_y_*, *E_y_* (eversion: +, inversion: −); and *G_z_*, *E_z_* (internal rotation: +, external rotation: −). GPC_a-d_ and GPC_α-θ_: specific significant predictors in IMS-measured foot motion, which will be described in Table 4 and in the Discussion. The results of p1 to p6 were cited from Ref. [20].

Figure 3 illustrates the ranges of the selected features of the GPCs. Table 3 provides an overview of the chosen GPs and IPAs in the estimation model for each knee indicator. Additionally, Table 3 details the GPCs shown in Figure 3.

For the knee joint angle indicators, two types of temporal indicators (p1 to p6), excluding p6, the SGA in the sagittal plane (*E_x_*) during the initial to mid-swing phases exhibited a small-to-medium-effect-sized positive correlation with the target variables. This suggests that the participants with higher plantarflexion angles during these phases had larger values for p1, p2, p3, and p6, as well as longer durations for p4 and p5. Furthermore, a reduction in the superior-direction acceleration during mid-swing increased p4 and p5. Notably, for p1, the SGA in the frontal plane (*E_y_*) during the early stance phases was selected as a significant feature. Similarly, for p6, the foot motion in the frontal plane during the swing phases was identified as a significant feature, with higher p6 values associated with a more everted foot posture during mid-swing.

For the AJC indicators (p7 to p10), *E_x_* was also significant, positively correlating with the target variable in most models (p7, p8, and p9). The participants with a higher *E_x_* in plantarflexion (p8, p9) or a lower *E_x_* in dorsiflexion (p7) exhibited a better AJC performance. The most significant GPCs for p8 and p9 during their respective regions of the GPC were *G_x_* during the mid-stance and *E_x_* during the terminal stance, displaying negative and positive correlations, respectively, with the target variables. This indicates that the participants with a lower foot rotation velocity and a higher SGA in the plantarflexion direction during these phases presented higher p8 and p9 values. Additionally, *E_x_* just before and after toe-off positively was correlated with p8 and p9, although not within the regions of the GPC. The foot motion in the frontal and horizontal planes (*G_y_* and *G_z_*) was significant for estimating p10, with a higher foot rotation velocity in the eversion direction during the latter terminal stance positively influencing p10.

Similar to p1 to p6, there was a positive correlation between age and the target variables. However, the model diverged by incorporating sex as a predictive factor for p7, p8, and p9, suggesting potential sex differences in the AJC. The stride time (GP_10_) demonstrated a negative correlation with all the AJC indicators, particularly showing medium to large effect sizes with p9 and p10 (*r* = −0.481, −0.534). Together with gait speed (GP_2_), which had a large effect size with p9 and p10 (*r* = 0.613, 0.602), these findings suggest that participants with shorter stride times and greater gait speeds have a superior AJC.

### 3.3. Model Evaluation

**Figure 4:**
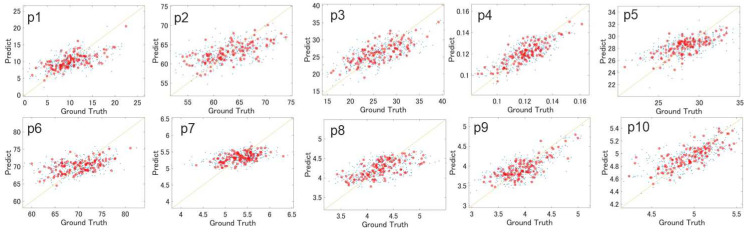
Agreement plots between true and model-predicted values of p1 to p10 of training. The small blue dots indicate the average data of every single walking trial. The big red dots indicate the average data of each subject. The results of p1 to p6 were cited from Ref. [20].

Figure 4 displays the agreement plots for p1 to p10 derived from the LOSOCV, and Table 5 consolidates the assessment indices for each model.

Upon evaluating the *R*^2^s and *r*s, all the models achieved medium to large effect sizes. In the evaluation of the ICC (2,1), the parameters from p1 to p5 and p8 to p10 attained a “fair” to “good” agreement. This indicates that the knee indicators, expressed as variations in the angle or the timing for daily walking gait behaviors, can be accurately estimated. In contrast, indicators p6 and p7 demonstrated a “poor” agreement. The lower agreement for p6 could be attributed to variance in the baseline calibration of each participant’s knee flexion angle during measurement. For p7, it could be due to noise in body movement caused by the displacement of sensors embedded in the pants during walking.

According to the results shown in Table 5, the mean predicted values yielded by the estimation models closely aligned with the average measured values, introducing an error of less than 1%. This consistency can be attributed to the use of a linear regression model combined with LOSOCV for constructing the models. However, the standard deviation of the predicted values tended to be lower than those of the measured values, suggesting that the predictability of the models slightly diminished for data points far from the mean value. When comparing the MAE and *M_t_*, the MAE confirmed that the estimated values fell within 0.54 to 0.75 times the standard deviation range of the measured values.

## 4. Discussion

### 4.1. Connection Between Knee and Foot Motion

In this study, we identified several noteworthy foot motion features relevant to the estimation of knee motion indicators, particularly the GPCs in SGAs, i.e., foot posture angles.

**Figure 5:**
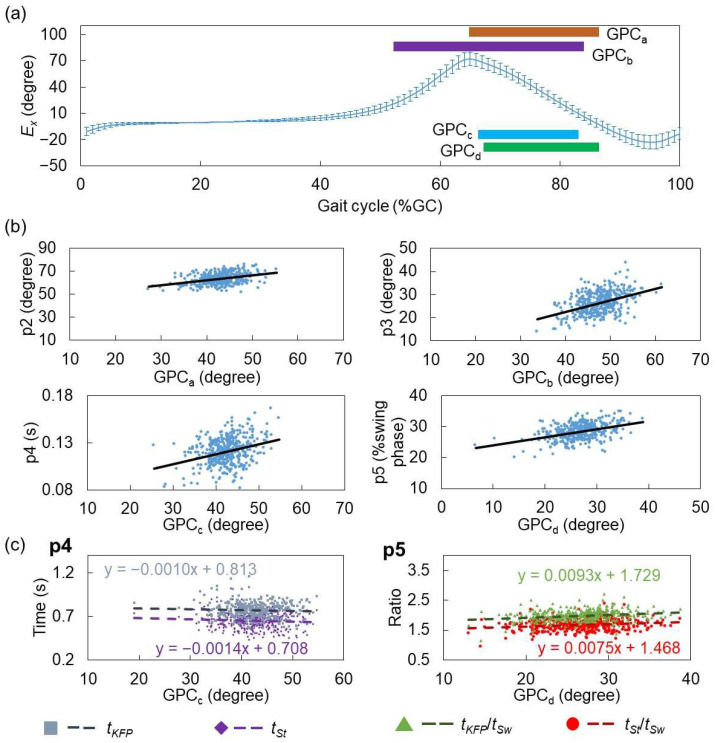
Analysis of the relationship between *E_x_* and two knee joint angle and temporal indicators. (**a**) Average and standard deviation of *E_x_* in different groups of subjects. The region of corresponding GPCs (see Figure 3) is also represented by different color bars. (**b**) Scatter plots of two knee joint angle and temporal indicators, p2 to p5, with their corresponding predictors from *E_x_*. (**c**) Scatter plots of GPC_c_ and GPC_d_ with the items in the formula for the p4 and p5 calculations (see Equation (1)). The dots and lines in different colors show the data and their tendencies.

Figure 5a exhibits the *E_x_* waveforms and the range of the identified GPCs of interest. The IMS measured the SGA in the sagittal plane (*E_x_*) during walking, influenced by both the angle of the ankle joint and the flexion/extension angle of the knee joint. Distinct segments of *E_x_*, from the terminal stance to mid-swing, were specifically selected as the principal predictors for the estimation of indicators p2 to p5, denoted as GPC_a_, GPC_b_, GPC_c_, and GPC_d_ (see Figure 3). Understanding the relationship between *E_x_* and these indicators is pivotal, forming the focus of this section. The relationship between each predictor and the corresponding indicator is presented in Figure 5b, showing that the values of p2 to p5 increase with the enhancement of amplitude.

At the end of the gait cycle, sufficient knee joint extension is required to prepare for landing. During this period, the ankle joint maintains a neutral position in the sagittal plane, and thus, *E_x_* is primarily determined by the degree of knee extension. A greater knee extension results in larger *E_x_* values in the dorsiflexion direction (negative by definition). Additionally, an increased knee extension generates more knee joint power, which raises the ground reaction forces (GRFs) upon landing, necessitating more knee joint flexion in the early stance phase (p1) to absorb the impact. Increased GRFs also cause more eversion (positive *E_y_*) torque at the subtalar joint during the early stance phase [33,34]. This correlation between p1 and GPC_α_ and GPC_β_ (Figure 3) can be explained in this context.

Previous research indicates that older participants demonstrate lower p2 values [35]. Our analysis also showed a negative correlation between age and p2 (*p* < 0.001), endorsing the credibility of our results. From the initial swing to mid-swing, the ankle joint begins to reduce plantarflexion, implying that foot motion—specifically, the amplitude of *E_x_*—should primarily be influenced by knee flexion. Similarly, the knee and ankle joints maintain neutral positions in the frontal plane, indicating that *E_y_*, representing foot posture in this plane, is mainly determined by hip abduction [33,36]. As knee flexion during the swing phase is mainly passive due to hip flexion and gastrocnemius muscle activity [37], the correlation between p6 and GPC_γ_ may indicate the influence of hip abductors, which work in conjunction with hip flexors during this gait phase.

Participants with stiff knees displayed a worse knee motion performance [38], affecting gait adversely. Goldberg et al. [19] and Ezaki et al. [39] suggested that participants with stiff knee tendencies have lower p2 values and longer p4 and p5 durations due to the late appearance of KFP during the swing phase. Deasy et al. [40] postulated that participants with knee OA often have weaker hip flexors, resulting in reduced lower limb elevation during the swing phase. This understanding leads to the inference that participants with reduced lower limb elevation during the swing phase likely exhibit elongated p4 and p5 timings. A peak signal in the superior direction can be observed in the *A_z_* waveform during the mid-swing phase [17]. The negative correlations between GPC_δ_ and GPC_ε_ with p4 and p5 (Figure 3) support this inference.

According to previous studies [19,39], participants with a poorer knee motion performance may exhibit longer durations for p4 and p5. Yaguchi et al. [12] indicated through a clinical study that individuals with deteriorating knees show decreased knee flexion and a decreased *E_x_* peak during the swing phase. This suggests the following: (1) participants with a worse knee motion performance may have lower GPC_c_ and GPC_d_ values; and (2) p4 and p5 should be negatively correlated with GPC_c_ and GPC_d_. However, contrary to this deduction, Figure 5b shows positive correlations between p4 and p5 with GPC_c_ and GPC_d_. To address this contradiction, we analyzed the relationship between *E_x_* and p4 and p5 in more detail. According to Equation (1), both p4 and p5 depend on *t_KFP_*, *t_St_*, and *t_Sw_*. The relationship between GPC_c_ and GPC_d_ with the elements constituting p4 and p5 is depicted in Figure 5c. For p4, both *t_KFP_* and *t_St_* decline with an increasing GPC_c_. Conversely, for p5, both *t_KFP_*/*t_Sw_* and *t_St_*/*t_Sw_* increase with an elevation in GPC_d_. Figure 5c demonstrates that the regression line gradient for *t_KFP_* and *t_KFP_*/*t_Sw_* is larger than for *t_St_ and t_St_/t_Sw_*, respectively, explaining the inconsistency. Combining this with the findings of Yaguchi et al. [12], our results suggest that, rather than focusing on p4 and p5, detecting the decline in *E_x_* signals during the early swing phase in daily gait monitoring may hold more potential for the early detection of knee deterioration. To validate this assumption, we will conduct a similar analysis on another group of participants with a higher age and knee OA in the next study.

All the AJC indicators were found to be negatively correlated with the stride time duration (GP_10_). Additionally, positive correlations were observed between p9 and p10 and gait speed, consistent with the findings by Fukaya et al. [41]. During the transition from the terminal stance to pre-swing, the calf muscles contract to rotate the foot from dorsiflexion to plantarflexion and from inversion to eversion [33]. Goldberg et al. [17] suggested that the gastrocnemius muscle positively affects the peak knee flexion velocity. Similarly, Fukaya et al. [41] proposed that knee angular acceleration and velocity undergo similar changes during the terminal stance and pre-swing phases. These previous studies may provide insight into the correlations observed between GPC_ζ_, GPC_η_, GPC_θ_, and AJC indicators p8 to p10 in our research.

### 4.2. Limitations of This Technology

Currently, our study is at the feasibility analysis stage. However, for practical daily applications, a real-time algorithm for assessing knee motion indicators is essential. Building upon our previous research, we have proposed online algorithms for estimating gait predictors for daily gait analyses using an IMU [11,15]. Leveraging these methodologies, we posit that routine knee health assessments can be achieved through the use of an IMU.

Despite our progress, this study has several limitations. During the model construction phase, we used sensor-embedded pants to measure lower limb kinematics based on IMU signal calculations. However, complications arose with the sensor-embedded pants. First, due to the limited range of available pants sizes, not all participants were adequately fitted, potentially hindering the precise positioning of the IMUs and subsequent kinematic data collection. Second, sensor displacement during walking was observed. These non-technical factors may have introduced noise, such as variance in the calibrated baselines of the knee flexion angle and body movement noise, affecting the accuracy of the model estimates for p6 and p7. It is conceivable that noise-cancellation technologies will be necessary during pre-processing when using wearable motion capture systems in future studies.

In this study, we only included younger and healthy participants to construct the estimation model. To expand the application to clinical use, the models must be improved by involving participants with conditions such as OA, muscle paralysis, or neuromuscular disorders. We plan to include another group of participants with a higher age and knee OA to improve the model in the next study.

## 5. Conclusions

In this study, we successfully developed models to estimate ten knee indicators for assessing knee motion impairment using IMS-obtained foot motion data from healthy participants through multivariate linear regression. Among the knee joint angle indicators, three out of four showcased a “fair” agreement, while one displayed a “poor” agreement. For the temporal indicators, one demonstrated a “good” agreement and the other a “fair” agreement. Regarding the AJC indicators, three out of four achieved a “fair” or “good” agreement, with one showing a “poor” agreement. Overall, eight out of the ten estimation models are promising candidates for application in daily living scenarios. The SGAs, particularly the foot posture angles in both the sagittal and frontal planes, emerged as significant predictors for each knee indicator. We demonstrated the possibility that knee indicators can be estimated from foot motion, even if the sensor is not attached to the knee directly. Looking forward, we intend to test these models on older healthy and OA participants to explore their broader applicability. We also aim to develop an application to assist with user rehabilitation, grounded in the insights derived from this study.

## Figures and Tables

**Table 1:** The results for the IMS-measured gait parameters.

	Mean ± SD
Data number	403
GP_1_ (m)	1.38 ± 0.15
GP_2_ (m/s)	1.30 ± 0.16
GP_3_ (°)	27.96 ± 5.35
GP_4_ (°)	72.59 ± 5.71
GP_5_ (cm)	3.15 ± 1.44
GP_6_ (cm)	14.02 ± 1.84
GP_7_ (°)	11.69 ± 5.39
GP_8_ (%GC)	62.71 ± 1.86
GP_9_ (%GC)	37.29 ± 1.86
GP_10_ (s)	1.06 ± 0.09

Standard deviation (SD), stride length (GP_1_), gait speed (GP_2_), maximum sole-to-ground angle (SGA) in dorsiflexion direction (GP_3_), SGA in plantarflexion direction (GP_4_), maximum circumduction (GP_5_), maximum foot height (GP_6_), toe in/out angle in the transverse plane (GP_7_), proportion of stance phase (GP_8_) and swing phase (GP_9_), and stride time (GP_10_).

**Table 2:** Pearson’s correlation coefficient between demographic data and p1 to p10.

	p1	p2	p3	p4	p5	p6	p7	p8	p9	p10
Sex	0.213 **	−0.247 **	−0.061	−0.386 **	−0.174 **	−0.083	0.554 **	0.824 **	0.229 **	0.136 *
Age	0.144 *	−0.441 **	−0.147 *	−0.489 **	−0.510 **	−0.483 **	0.422 **	0.136 *	0.334 **	0.397 **
BMI	−0.187 **	0.074	0.160 *	−0.016	0.140 *	−0.136 *	−0.083	−0.129 *	−0.073	0.191 **

*: significance level, *p* < 0.05; **: significance level, *p* < 0.001.

**Table 3:** The selected IPA and GP predictors in different models.

Model No.	Selected IPA and GP Predictors
p1	**GP_1_**, **GP_3_**, **GP_8_**, **Age**
p2	**GP_6_**, **GP_10_**, **Age**
p3	**GP_1_**, **GP_3_**, **GP_6_**, **GP_7_**, **Weight**
p4	**GP_1_**, **GP_3_**, **GP_6_**, **GP_7_**, **GP_8_**, **GP_10_**, **Age**, **Height**
p5	**GP_1_**, **GP_7_**, **GP_8_**, **Age**, **Weight**
p6	**GP_1_**, **GP_4_**, **GP_5_**, **Age**
p7	**GP_1_**, **GP_4_**, **GP_6_**, **GP_10_**, **Sex**, **Age**, **Weight**, **Height**
p8	**GP_7_**, **GP_10_**, **Sex**, **Age**, **Weight**, **Height**
p9	**GP_1_**, **GP_2_**, **GP_3_**, **GP_4_**, **GP_5_**, **GP_6_**, **GP_8_**, **GP_10_**, **Sex**, **Age**
p10	**GP_2_**, **GP_4_**, **GP_6_**, **GP_10_**, **Age**, **Weight**

Texts in dark blue: *r* ≤ −0.500 (large effect size). Text in medium blue: -0.499 < *r* ≤ −0.300 (medium effect size). Texts in light blue: −0.299 < *r* ≤ −0.100 (small effect size). Texts in yellow: −0.100 < *r* < 0.100 (none). Texts in light red: 0.100 ≤ *r* < 0.300 (small effect size). Texts in medium red: 0.300 ≤ *r* < 0.500 (medium effect size). Texts in dark red: *r* ≥ −0.500 (large effect size). IPA: individual physical attributes, including sex, age, weight and height; stride length (GP_1_), gait speed (GP_2_), maximum sole-to-ground angle (SGA) in dorsiflexion direction (GP_3_), maximum SGA in plantarflexion direction (GP_4_), maximum circumduction (GP_5_), maximum foot height (GP_6_), and toe in/out angle in the transverse plane (GP_7_), proportion of stance phase (GP_8_) and swing phase (GP_9_), and stride time (GP_10_). The results of p1 to p6 were cited from Ref. [20].

**Table 4:** The description of foot motion in GPCs depicted in Figure 3.

GPC No.	Descriptions
GPC_a-d_	SGA in plantarflexion direction during pre-swing to mid-swing.
GPC_α_	SGA in dorsiflexion direction during terminal swing.
GPC_β_	SGA in frontal plane during loading response and mid-stance.
GPC_γ_	SGA in eversion direction during mid-swing.
GPC_δ_	Foot acceleration in superior direction during mid-swing.
GPC_ε_	Foot acceleration in superior direction during mid-swing.
GPC_ζ_	SGA in plantarflexion direction during terminal stance to initial swing.
GPC_η_	SGA in plantarflexion direction during terminal stance to initial swing.
GPC_θ_	Foot rotation velocity in eversion direction during terminal stance.

Age was factored into the model construction for all the indicators except p3. The stride length (GP_1_) was included in the model construction for all the indicators except p2. Although the stride length demonstrated opposing correlations to p1 and p5, the correlation coefficients were very low (age in p1: *r* = 0.083; GP_1_ in p5: *r* = −0.065). The maximum SGA in the dorsiflexion direction (GP_3_) was selected for models p1, p3, and p4, with larger GP_3_ values associated with larger p1, p3, and p4 values. Sex was not selected as a predictive factor for p1 to p6.

**Table 5:** The results of the model evaluation.

	p1	p2	p3	p4	p5	p6	p7	p8	p9	p10
***R*** **^2^**	0.400	0.369	0.475	0.607	0.376	0.255	0.182	0.352	0.513	0.515
**ICC**	0.489	0.473	0.594	0.712	0.482	0.335	0.245	0.442	0.611	0.619
***r***	0.540	0.535	0.635	0.734	0.543	0.415	0.329	0.502	0.651	0.657
**MAE**	2.65	3.13	2.88	0.0076	1.73	3.00	0.27	0.27	0.23	0.16
***M_t_***	10.20	63.09	26.22	0.121	28.28	70.17	5.34	4.27	3.94	4.95
***SD_t_***	4.03	4.67	4.62	0.0140	2.54	4.16	0.36	0.40	0.37	0.27
***M_e_***	10.19	63.08	26.20	0.121	28.27	70.16	5.35	4.27	3.94	4.94
***SD_e_***	2.56	2.81	3.13	0.0109	1.54	2.10	0.16	0.24	0.26	0.19

*R*^2^: coefficient of determination of the models; ICC: type (2, 1) of the intra-class correlation coefficient between true and estimated values; *r*: Pearson’s correlation coefficient between true and estimated value. Units of MAE: degree (p1, p2, p3, p6); s (p4); %swing phase (p5); log(rad^2^/s^5^) (p7 to p10). *M_t_* and *M_e_*: mean values of true and estimated values; *SD_t_* and *SD_e_*: standard deviations of true and estimated values. The results of p1 to p6 were cited from Ref. [20].

## Data Availability

The datasets presented in this article are not readily available due to privacy, legal, or ethical reasons.
